# Phenotypic and genotypic characterization of *Enterococcus faecalis* and *Enterococcus faecium* isolated from fish, vegetables, and humans

**DOI:** 10.1038/s41598-024-71610-0

**Published:** 2024-09-18

**Authors:** Asmaa Gaber Mubarak, Mona Ahmed El-Zamkan, Waleed Younis, Sahar Osman Saleh, Hanan H. Abd-Elhafeez, Asmaa Gahlan Yoseef

**Affiliations:** 1 Department of Zoonoses, Faculty of Veterinary Medicine, South Valley University, Qena, 83523 Egypt; 2 Department of Food Hygiene and Control, Faculty of Veterinary Medicine, South Valley University, Qena, 83523 Egypt; 3 Department of Microbiology, Faculty of Veterinary Medicine, South Valley University, Qena, 83523 Egypt; 4 Department of Cell and Tissues, Faculty of Vet. Medicine, Assiut University, Assiut, 71526 Egypt

**Keywords:** Biofilm, *Enterococcus* species, Resistance, Sequence, Virulence, Genetics, Microbiology, Pathogenesis

## Abstract

Enterococci, common hospital-acquired infections in immunocompromised patients, have garnered attention in clinical microbiology. To determine the clinical relevance of enterococci as food-borne pathogens, 116 fish, 90 vegetables, and 120 human diarrheal samples were tested for *E. faecalis* and *E. faecium* pathogenicity. Conventionally, 69 of 326 (21.17%) samples were positive for *Enterococcus* species, 52 (15.95%) of which were molecularly classified as *E. faecalis* and 13 (3.99%) as *E. faecium*. The *E. faecalis* contamination percentage of fresh fish (19.70%) was higher than frozen fish (4%). Cauliflower had the highest *E. faecalis* percentage (16.67%) when fish and vegetable samples didn’t harbor the *E. faecium atpA* gene. 23.33% and 10.83% of participants’ samples were molecularly confirmed as *E. faecalis* and *E. faecium* positive, respectively. *E. faecalis* isolates had all virulence genes, with *gel*s being the most common (65.38%), while *cylA* and *asa1* genes couldn’t be detected in *E. faecium* isolates. *E. faecalis* showed the highest resistance against vancomycin and tetracycline (69.23%), whereas *E. faecium* extremely resisted tetracycline (76.92%) and erythromycin (69.23%) with the recognition of MDR among 44.2% of *E. faecalis* and 38.5% of *E. faecium* isolates. The great similarity of our isolates showed the clinical importance of food-borne antibiotic-resistant enterococci.

## Introduction

*Enterococcus* are Gram-positive, facultative anaerobic bacteria resistant to harsh environmental conditions, so the microorganism is widely distributed in soil, food, feed, sewage, and freshwater, besides the gastrointestinal tracts of both animals and humans^1^. Genus *Enterococcus* consists of more than 50 species, of which *E. faecalis* and *E. faecium* are accountable for human infections at 80–90% and 5–15%, respectively^2,3^. *Enterococcu*s are robust organisms that tolerate various temperatures and pH levels and display better environmental endurance.

Enterococci have been largely identified in several foods of animal origin, including vegetables^4,5^. In addition, *Enterococcus* species is a commensal bacterium in seafood that gets contaminated from numerous sources, such as lakes, rivers, and seawater, as they tolerate high salt concentrations and can survive longer in marine environments^6,7^. Fish farming is one of the best ways to combat overuse of natural resources. Most people rely on fish as their primary source of animal protein, making fish an essential part of food globally. However, fish waste may hurt the local ecosystem by polluting the water and land^8^. The second most widely cultivated fish in the world, after carp, is tilapia, which grown extensively in Egypt^9^ with *E. faecalis* pathogenicity ranging from high, moderate, to low. Enterococcosis, sometimes called “pop-eye disease,” is an infectious fish disease with a high aquarium mortality rate that causes financial losses^10^.

Vegetables may act as a source of various pathogenic bacteria, especially enterococci, which get contaminated through wastewater used in irrigation or the use of organic fertilizers, as this microbe remains in the soil for up to 100 days^11^. The main transmission method of enterococci to humans is through the handling and consuming contaminated food^12^, but direct transmission may also occur through contact with animals or their environment. So, veterinarians, farmers, and slaughterhouse workers are at high risk of enterococcal infection. Dissemination may also arise in hospitals by healthcare workers and inanimate objects^13^.

Despite being regarded as harmless commensals, *Enterococcus* species, in particular *E. faecalis,* followed by *E. faecium,* play an important role in intensive care units, where they have emerged as a global source of nosocomial infections, especially in immunocompromised patients and those who have serious underlying diseases^14,15^. They can also cause gastrointestinal infections, including diarrhea, especially in the elderly and children^16^.

Enterococci pathogenesis is ascribed to a variety of virulence factors that can be categorized into two groups: secreted virulence factors such as hyaluronidase (*hyl*), gelatinase (*gelE*), cytolysin (*cylA*), and cell surface virulence factors comprising enterococcal surface protein (*esp*), aggregation substances (*asa1*), collagen-binding protein (*ace*), and endocarditis antigen (*efaA*) which play an important role in biofilm formation and adhesion to host cells^17^.

*E. faecalis* and *E. faecium* attract more attention due to their substantial resistance to multiple antibiotics through plasmid and transposon transfer, chromosomal exchange, or mutations^18^. Due to the noticeable plasticity of their genomes, *E. faecalis* and *E. faecium* can acquire new resistance determinants. Particularly vancomycin-resistant strains, which have become a major nosocomial bacteria worldwide^19^.

Vancomycin-resistant enterococci (VRE) was stated by WHO as “high priority”^20^ and recently, the significance of alternative antibiotics like linezolid (LZD) has recently grown in response to the emergence of vancomycin-resistant enterococci. Linezolid is an oxazolidinone that binds to rRNA on both the 30S and 50S ribosomal subunits to prevent bacteria from synthesizing proteins^21^. Enterococci’s resistance to antibiotics leads to infections in children, the elderly, immunocompromised people, and cancer patients. Infected patients with diarrhea can be the source of MDR-enterococci, especially vancomycin^16^.

One of Enterococcus’ most obvious pathogenicity traits is biofilm production, an assemblage of surface-associated microbial cells enclosed in an extracellular polymeric substance matrix. Biofilms facilitate adhesion to host cells and colonize inert and biological surfaces. Generally, biofilm formation protects bacteria from phagocytosis, antibiotics, and host immunological responses^22^. Over 80% of enterococci-related illnesses in the U.S.A. are associated with slime production^23^. So, this study describes the isolation and identification of *E. faecalis* and *E. faecium* from fish, vegetables, and diarrheal samples, as well as antimicrobial resistance profiles, biofilm formation, and virulence of the obtained isolates.

## Results

### Occurrence of *Enterococcus* species in food samples

As shown in Table 1, sixty-nine out of 326 (21.17%) investigated samples were positive for *Enterococcus* species based on a cultural and biochemical examination, of which 52 (15.95%) and 13 (3.99%) were identified molecularly as *E. faecalis* and *E. faecium,* respectively (Additional File 1, Figures S1 and S2). Among fresh fish samples, tilapia harbored a higher contamination rate of enterococci 9 (21.95%) than catfish 5 (20%). The *E. faecalis 16S rRNA* gene was detected through molecular confirmation in 8 (19.51%) of tilapia isolates, while all the obtained catfish isolates (5, 20%) harbored the gene. Two of the four enterococci isolates from frozen fish were identified as *E. faecalis* positive (4%). As for the examined 90 vegetable samples, *E. faecalis* could be detected with the acquisition of cauliflower at 16.67%, followed by equal percentages in parsley and lettuce at 6.67%. The presence of *E. faecalis* in each of fish (*P* = 0.043) and vegetables (*P* = 0.016) is significantly correlated with its occurrence in human samples (*P* < 0.05), while higher significance (*P* = 0.007) existed between food (both fish and vegetables) and human isolates. Molecular assessment has revealed that the investigated food samples, including fish and vegetables, are free from *E. faecium*. At the same time, it was discovered that four isolates didn’t harbor *E. faecalis 16S rRNA* or *E. faecium atpA* genes, which are suspected to be other *Enterococcus* species.

**Table 1:** Occurrence of *Enterococcus* species in the examined samples.

Sources of examined samples		No. of examined samples	Conventional methods	PCR method		
			+ ve Enterococci No. (%)	*E. faecalis* No. (%)	*E. faecium* No. (%)	Other Enterococcus species
Fresh fish						
Tilapia		41	9 (21.95)	8 (19.51)	0 (0)	1(2.44)
Catfish		25	5 (20)	5 (20)	0 (0)	0 (0)
Sub-Total		66	14 (21.21)	13 (19.70)	0 (0)	1 (1.51)
Frozen fish		50	4 (8)	2 (4)	0 (0)	2 (4)
Total		116	18 (15.52)	15 (12.93)*	0 (0)	3 (2.59)
Vegetables						
Parsley		30	3 (23.33)	2 (6.67)	0 (0)	1 (3.33)
Lettuce		30	2 (6.67)	2 (6.67)	0 (0)	0 (0)
Cauliflower		30	5 (16.67)	5 (16.67)	0 (0)	0 (0)
Total		90	10 (11.11)	9 (10)*	0(0)	1 (1.11)
Human		120	41 (34.17)	28 (23.33)*	13 (10.83)	0 (0)
Over all total		326	69 (21.17)	52 (15.95)	13 (3.99)	4 (1.23)

*Significant relation between each of total fish and human diarrheal isolates & total vegetables and human isolates (*P* < 0.05).

### Occurrence of *Enterococcus* species in human samples and risk factors assessment

In the present research, 41 enterococci isolates (34.17%) were obtained from 120 diarrheal samples collected from patients admitted to clinical laboratories in Qena Governorate with gastrointestinal issues. All isolates were assessed using PCR to characterize both species. A higher infection rate with *E. faecalis* (23.33%) than with *E. faecium (*10.83%) was detected in the patients (Table 1).

**Fig. 1:**
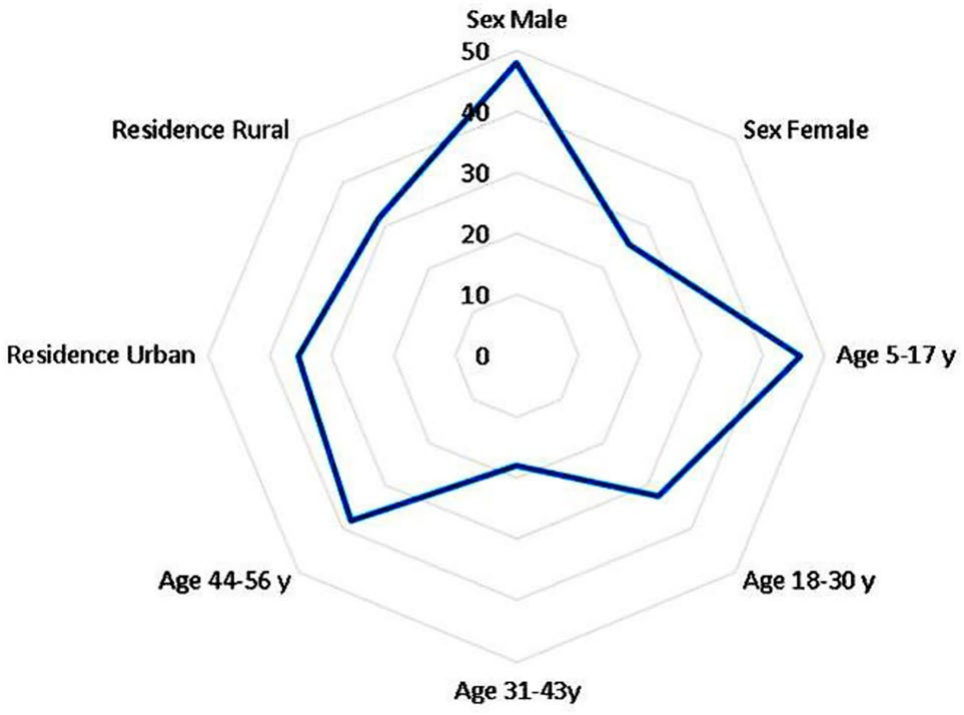
Occurrence of *Enterococcus* species in humans related to the associated risk factors.

The infection rate differed, with 47.83% among 46 male patients in contrast to 25.68% in 74 females. Regarding participants’ age, which ranged from 5 up to 56 years, the younger age group (5–17 years) showed the highest rate of enterococci infection (45.95%), followed by elderly patients (44–56 years) at 38.10%. The 18–30 age group had a 32.35% infection rate, while the lowest percentage (17.86) was for patients aged between 31 and 43 years. The distribution of residents was 79 patients from urban areas and 41 from rural areas; the results revealed an insignificant association between the infection rate among urban residents (35.44%) and rural (31.71%) (Fig. 1).

### Phylogenetic analysis

**Fig. 2:**
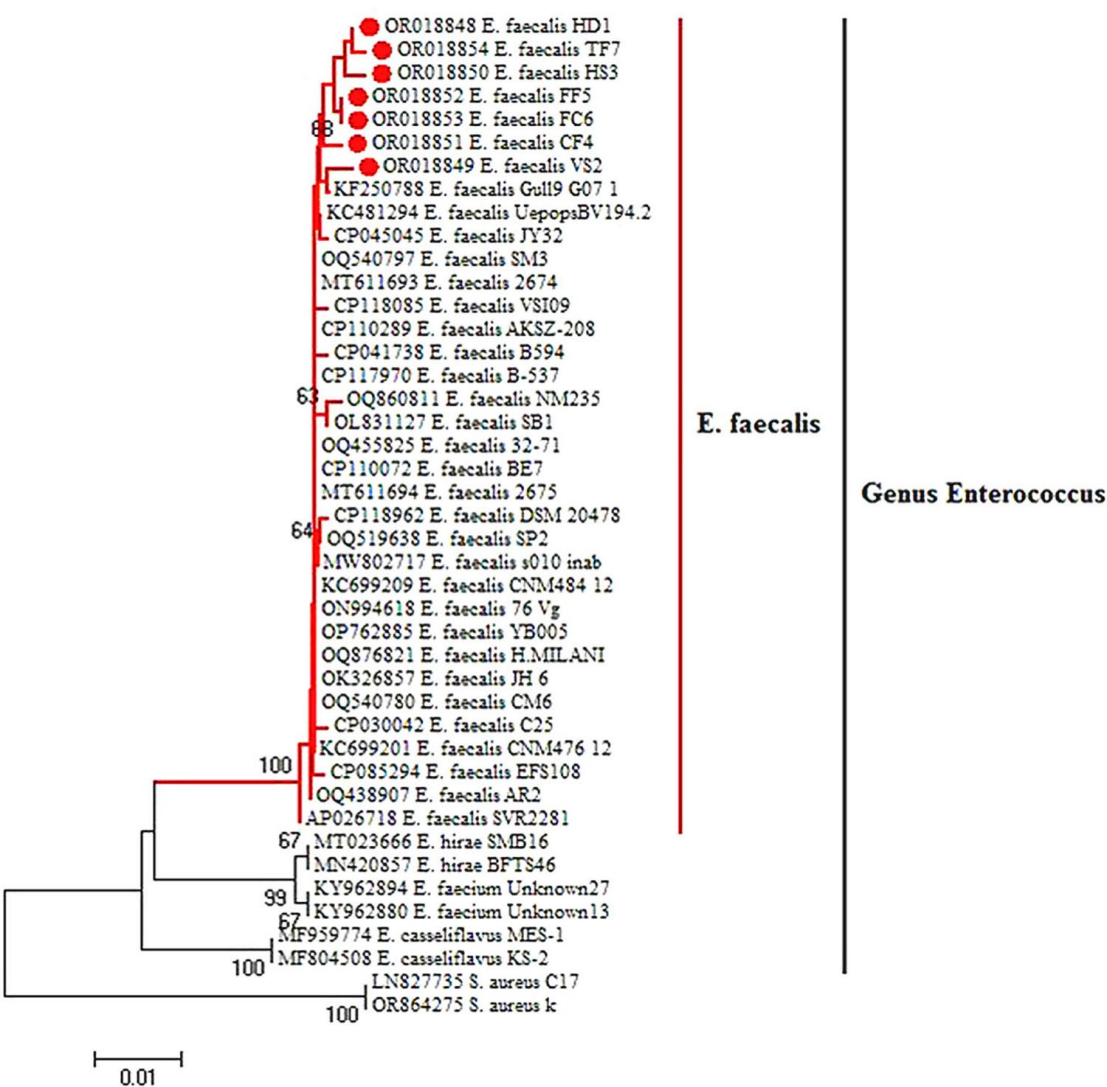
Neighbor-joining phylogenetic tree based on *16 S rRNA* gene sequence showing the relationship between the obtained *E. faecalis* isolates from fish, vegetables, and humans and the phylogenetically related reference strains on GenBank.

*16SrRNA* sequencing for seven randomly selected *E. faecalis* isolates from tilapia fish (OR018854 *E. faecalis* TF7), catfish (OR018851 *E. faecalis* CF4), frozen fish (OR018852 *E. faecalis* FF5 and OR018853 *E. faecalis* FC6), vegetables (OR018849 *E. faecalis* VS2), and diarrhea (OR018848 *E. faecalis* HD1 and OR018850 *E. faecalis* HS3) were performed, proving that our isolates were indeed *E. faecalis*. The neighbor-joining tree created by using the *16SrRNA* gene sequences revealed that our isolates were closely related to each other through sharing a common ancestor, with the somewhat exception of OR018849 *E. faecalis* VS2, which lodged in a more recent ancestor. The tree also showed high degrees of similarity between our isolates and those imported from the Genbank (Fig. 2).

### Occurrence of some virulence genes in *Enterococcus* isolates

**Fig. 3:**
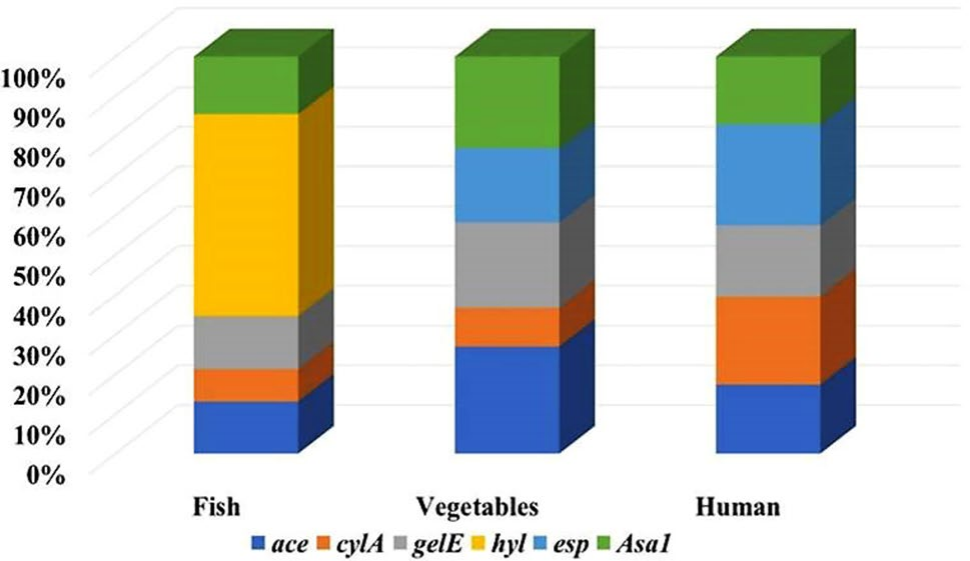
Distribution of virulence genes in *E. faecalis* among the examined samples.

**Fig. 4:**
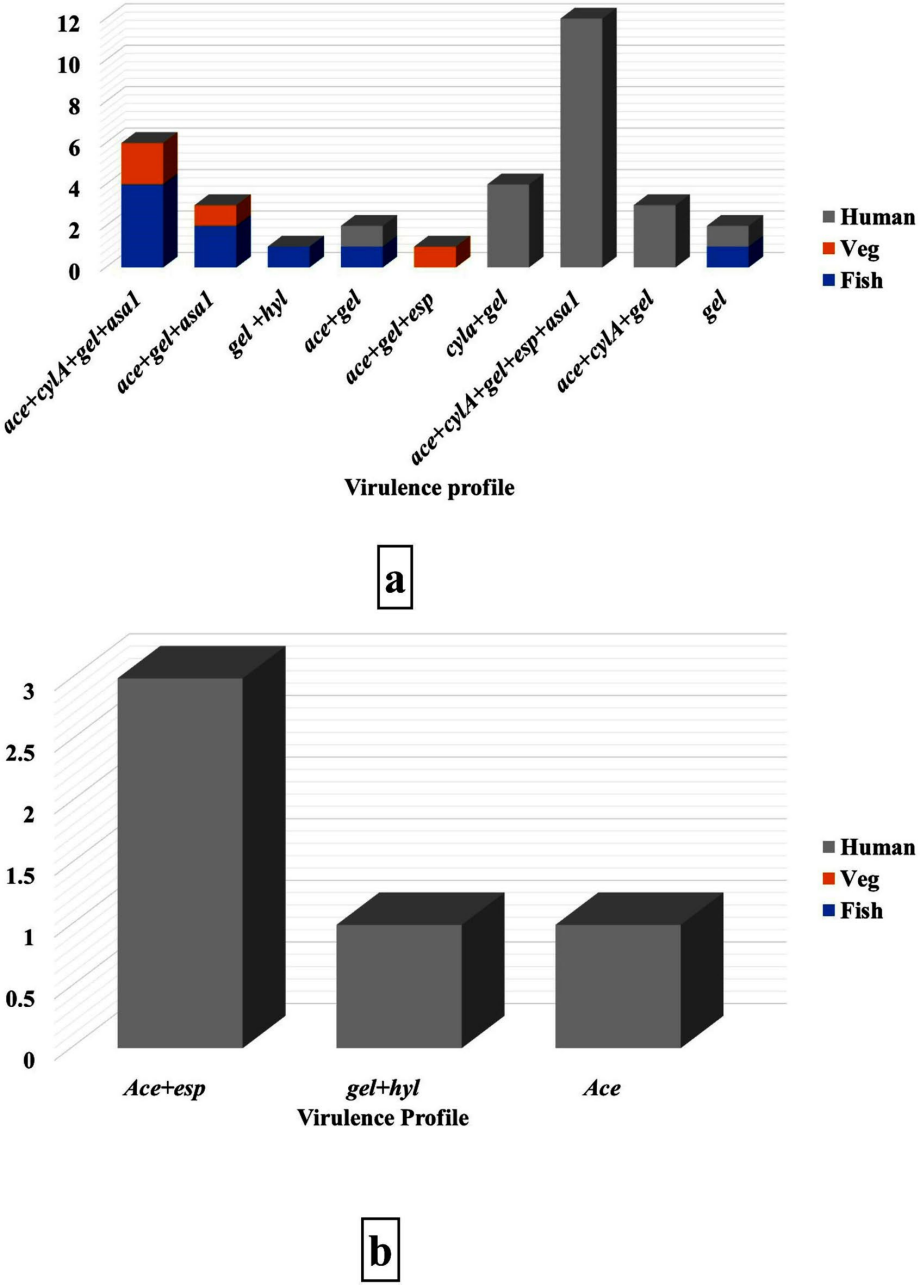
Different virulence gene profiles in *E. faecalis* (**a**) and *E. faecium* (**b**) among the examined samples.

The present study has assessed the occurrence of enterococcal virulence genes *ace, cylA, gelE, hyl, esp, and asa1* in the obtained isolates, as shown in Table 2 and Additional File 1, Figures S3–S8. The *gelE* gene was the most frequent in *E. faecalis* isolates (65.38%), followed by *ace* (51.92%), *cylA* (48.08%), *asa1* (40.38%), and *esp* (25%), while the least frequent one was *hyl* (1.92%). In general, the incidence of virulence genes was the highest in human isolates, followed by those obtained from fish. On the other hand, *E. faecium* obtained from patients’ samples showed a high percentage of *ace* gene (30.77%), followed by *esp* (23.08%), *gelE*, and *hyl* (7.69% for each) at a time when *cylA* and* asa1* genes couldn’t be detected*.* There is a significant correlation between the incidence of *cylA*, *gelE,* and *esp* in human isolates and their presence in food isolates (*P* < 0.05). The distribution of* asa1, gelE,* and *ace* genes *in E. faecalis* obtained from fish samples was nearly similar, while in strains isolated from vegetables, the *ace* gene was the highest, followed by *asa1* and *gelE*. On the other hand, *esp* was the most predominant gene, followed by *cylA,* in isolates of human origin (Fig. 3). The highest virulence profile was *ace* + *cylA* + *gel* + *esp* + *asa1*, which existed in human isolates, followed by *ace* + *cylA* + *gel* + *asa1*, which was detected in fish and vegetable isolates (Fig. 4a), while the most prevalent virulence profile in *E. faecium* isolated from human samples was *ace* + *esp* (Fig. 4b).

**Table 2:** Occurrence of virulence genes in *E. faecalis* and *E. faecium* isolates.

Sources of isolates No. of isolates	Ace No (%)	CylA* No (%)	gelE* No (%)	Hyl No (%)	Esp* No (%)	Asa1 No (%)
	*E. faecalis*					
Fish (15)	7 (46.67)	4 (26.67)	9 (60)	1 (6.67)	0 (0)	6 (40)
Vegetables (9)	3 (33.33)	1 (11.11)	3 (33.33)	0 (0)	1 (11.11)	2 (22.22)
Sub-total (24)	10 (41.67)	5 (20.83)	12 (50)	1 (4.17)	1(4.17)	8(33.33)
Human (28)	17 (60.71)	20 (71.43)	22 (78.57)	0 (0)	12 (42.86)	13 (46.43)
Total (52)	27 (51.92)	25(48.08)	34 (65.38)	1 (1.92)	13 (25)	21 (40.38)
	*E. faecium*					
Fish (0)	0 (0)	0 (0)	0 (0)	0 (0)	0 (0)	0 (0)
Vegetables (0)	0 (0)	0 (0)	0 (0)	0 (0)	0 (0)	0 (0)
Human (13)	4 (30.77)	0 (0)	1 (7.69)	1 (7.69)	3 (23.08)	0 (0)
Total (13)	4 (30.77)	0 (0)	1 (7.69)	1 (7.69)	3 (23.08)	0 (0)

*Significant relation (*P* < 0.05) between food and human isolates.

### Antibiotic resistance profile of *Enterococcus* isolates

Table 3 displays the antimicrobial resistance profile among both species of enterococci. Overall, 52 *E. faecalis* isolates were resistant to one or more antimicrobial agents, with vancomycin and tetracycline showing the highest resistance (69.23%), followed by erythromycin (61.54%). Chloramphenicol and nitrofurantoin resistance were detected in 57.69% of isolates, with moderate resistance to ciprofloxacin and linezolid at 30.77 and 40.38%, respectively. Ampicillin showed the highest activity against *E. faecalis* isolates; only three were resistant (5.77%). It is worth noting that all *E. faecalis* isolates of human origin showed complete susceptibility to ampicillin. In our study, *E. faecium* isolates could only be identified in patients’ diarrheal samples, which showed the highest resistance to tetracycline (76.92%) and erythromycin (69.23%). The same resistance was exhibited to ampicillin, vancomycin, and ciprofloxacin (46.15%). At the same time, linezolid was the most effective antibiotic against *E. faecium*, to which only 3 isolates (23.08%) resisted, followed by chloramphenicol and nitrofurantoin in a percentage of 38.46.

**Table 3:** Antimicrobial resistance profiles among *E. faecalis* and *E. faecium* isolated from different types of samples.

Samples	AMP No. (%)	VAN No. (%)	ERY No. (%)	TET No. (%)	CIP No. (%)	CHL No. (%)	LZ No. (%)	NIT No. (%)
Fish								
*E. faecalis* (15)	1 (6.67)	9 (60)	13 (86.67)	7 (46.7)	6(40)	7(46.67)	7(46.67)	9(60)
*E. faecium* (0)	0(0)	0(0)	0(0)	0(0)	0(0)	0(0)	0(0)	0(0)
Vegetables								
*E. faecalis* (9)	2(22.22)	7(77.78)	6(66.67)	7(77.78)	4(44.44)	4(44.44)	4(44.44)	4(44.44)
*E. faecium* (0)	0(0)	0(0)	0(0)	0(0)	0(0)	0(0)	0(0)	0(0)
Human								
*E. faecalis* (28)	0	20(71.43)	13(46.43)	22(78.57)	6(21.43)	19(67.86)	12(42.86)	17(60.71)
*E. faecium* (13)	6(46.15)	6(46.15)	9(69.23)	10(76.92)	6(46.15)	5(38.46)	3(23.08)	5(38.46)
Total								
*E. faecalis* (52)	3(5.77)	36(69.23)	32(61.54)	36(69.23)	16(30.77)	30(57.69)	21(40.38)	30(57.69)
*E. faecium* (13)	6(46.15)	6(46.15)	9(69.23)	10(76.92)	6(46.15)	5(38.46)	3(23.08)	5(38.46)

*AMP* ampicillin, *VAN* vancomycin, *ERY* erythromycin, *TET* tetracycline, *CIP* ciprofloxacin, *CHL* chloramphenicol, *LZ* linezolid, *NIT* nitrofurantoin.

**Fig. 5:**
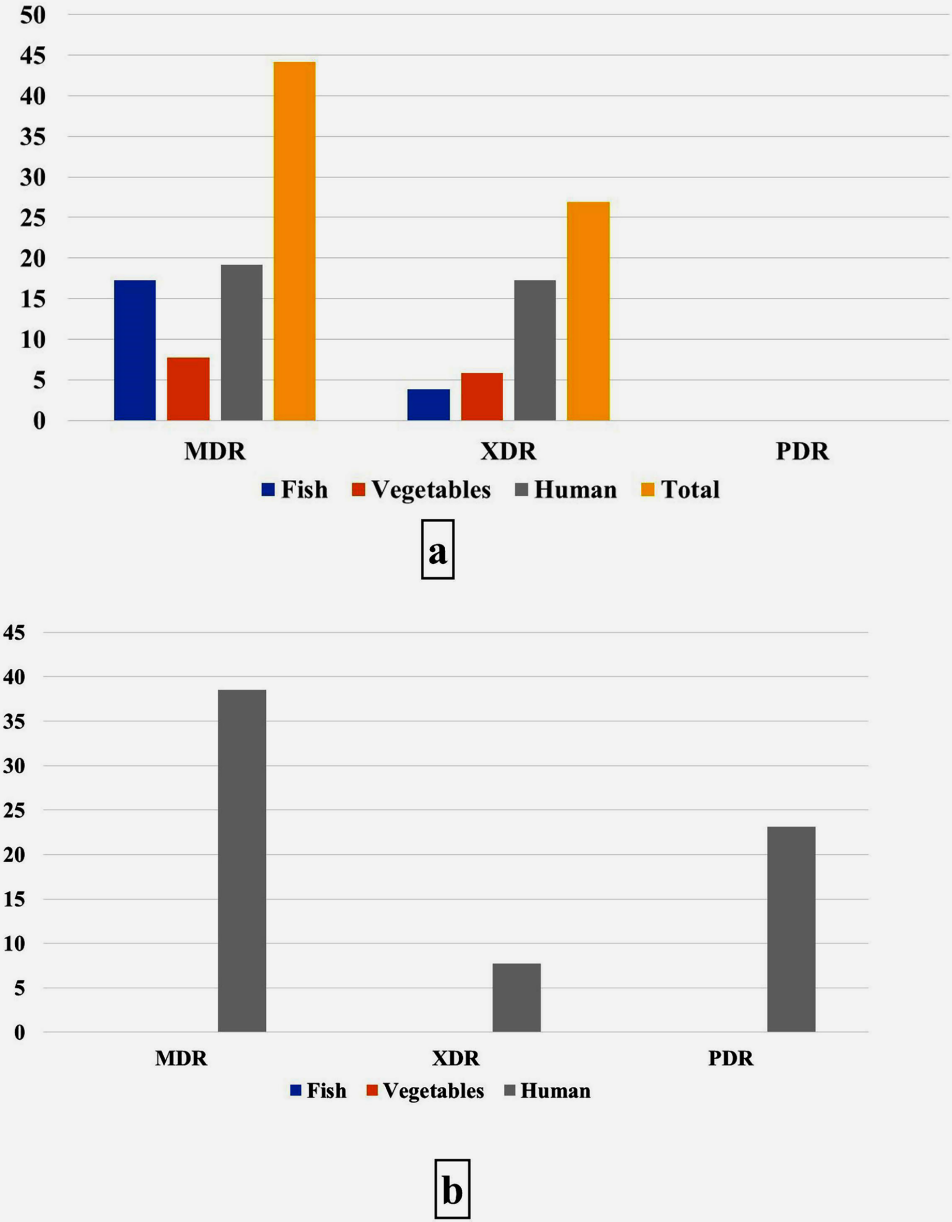
Incidence of M.D.R., XDR, and P.D.R. in *E. faecalis* (**a**) and *E. faecium* (**b**) isolates in different samples.

**Fig. 6:**
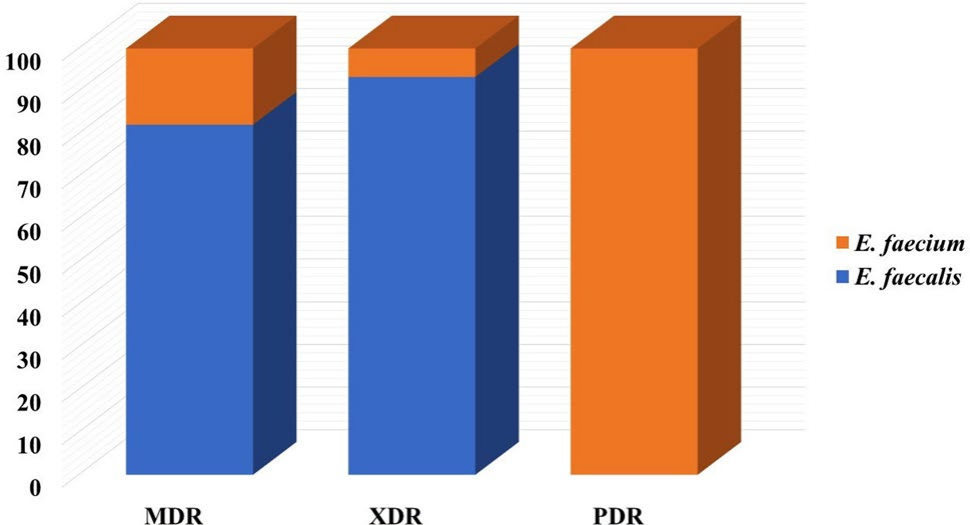
Distribution of M.D.R., XDR, and P.D.R. in *E. faecalis* and *E. faecium* isolates.

Data displayed in Table 4 revealed the presence of 26 different antimicrobial resistance patterns, of which *E. faecalis* and *E. faecium* exhibited 21 and 6 patterns, respectively. Three *E. faecium* isolates show resistance to all antibiotics used in this study. Figure 5a shows the incidence of MDR, XDR, and PDR strains; 44.2% of *E. faecalis* isolates show resistance to three or more antimicrobial categories (MDR), in which higher resistance levels were detected among human and fish isolates than vegetable, whereas 26.9% of the isolates were categorized as XDR, the with the highest incidence in vegetables isolates. MDR was recognized among *E. faecium* in the percentage of 38.5%, followed by PDR in 23.1% of isolates (Fig. 5b). Most MDR and XDR were among *E. faecalis* isolates, while PDR only appeared in *E. faecium* isolates (Fig. 6).

**Table 4:** Antimicrobial resistance patterns of *E. faecalis* and *E. faecium* isolated from different types of samples.

Antimicrobial resistance pattern	*E. faecalis (No = 47) No (%)	*E. faecium (No = 10) No (%)
AMP + VAN + ERY + TET + CHL	2 (4.3)	–
VAN + ERY + CIP + CHL + NIT	1 (2.1)	–
ERY + TET + CIP + LZ + NIT	2(4.3)	–
VAN + ERY + TET + CHL + LZ + NIT	2(4.3)	–
VAN + ERY + CIP + LZ + NIT	3 (6.4)	–
VAN + ERY	3 (6.4)	–
ERY + TET + CHL	2 (4.3)	–
CHL + NIT	1 (2.1)	–
AMP + VAN + ERY + TET + CIP + CHL	1 (2.1)	1 (10)
VAN + ERY + TET + CIP + CHL + LZ + NIT	4 (8.5)	–
VAN + TET + LZ	2 (4.3)	–
ERY + TET + CIP + NIT	1 (2.1)	–
TET + CHL	1 (2.1)	–
VAN + ERY + TET + CHL + NIT	5 (10.6)	–
VAN + ERY + TET + CHL + LZ + NIT	3 (6.4)	–
ERY + TET + LZ	3 (6.4)	–
VAN + CHL	2 (4.3)	–
VAN + TET + CHL + NIT	2 (4.3)	–
VAN + TET + CIP + CHL + LZ + NIT	4 (8.5)	–
VAN + TET	2 (4.3)	–
AMP + VAN + ERY + TET + CIP + CHL + LZ + NIT	–	3 (30)
ERY + TET + CIP	–	2 (20)
AMP + VAN + ERY + TET + NIT	–	2 (20)
ERY + TET + CHL	–	1 (20)
TET	–	1 (10)
NIT	1 (2.1)	–

*Five and three *E. faecalis* and *E. faecium* isolates were respectively sensitive to all the tested antimicrobials.

### Biofilm formation by *Enterococcus* isolates

Out of 52 obtained *E. faecalis* isolates, 22 (42.31%), 8 (15.38%), 3 (5.77%), and 19 (36.54%) were differentiated into strong, moderate, weak, and non-biofilm producers, noting that the highest percentages were for human isolates. On the other hand, more than half of the human *E. faecium* isolates (53.85%) couldn’t produce biofilm, while 4 (30.77%) and 2 (15.38%) were strong and moderate biofilm producers, respectively (Table 5).

**Table 5:** Biofilm formation by *E. faecalis* and *E. faecium* isolates.

Biofilm type	*E. faecalis* (No = 52)				*E. faecium* (No = 13)			
	Fish No. (%)	Vegetables No. (%)	Human No. (%)	Total No. (%)	Fish No. (%)	Vegetables No. (%)	Human No. (%)	Total No. (%)
Strong	7 (13.46)	4 (7.69)	11 (21.15)	22 (42.31)	0 (0)	0 (0)	4 (30.77)	4 (30.77)
Moderate	2 (3.85)	1 (1.92)	5 (9.62)	8 (15.38)	0 (0)	0 (0)	2 (15.38)	2 (15.38)
Weak	2 (3.85)	1 (1.92)	0 (0)	3 (5.77)	0 (0)	0 (0)	0 (0)	0 (0)
Non biofilm producer	4 (7.69)	3 (5.77)	12 (23.08)	19 (36.54)	0 (0)	0 (0)	7 (53.85)	7 (53.85)

### The correlation between drug resistance, biofilm formation, and presence of virulence genes

**Fig. 7:**
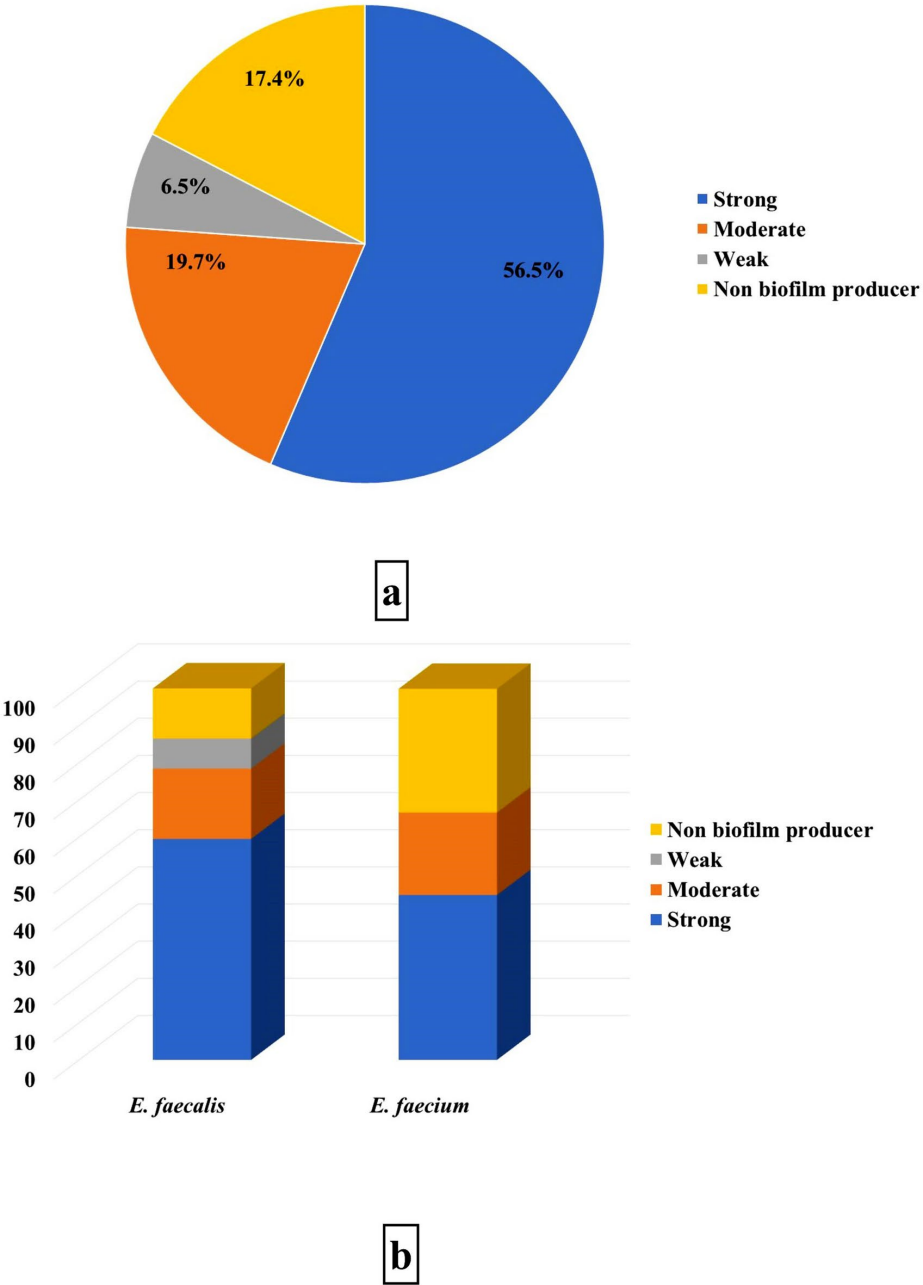
Biofilm formation by drug-resistant Enterococcus isolates. (**a**) Biofilm phenotype distribution among M.D.R., XDR, and P.D.R. Enterococcus isolates; (**b**) Biofilm phenotype distribution among M.D.R., XDR, and P.D.R. isolates with reference to species.

**Fig. 8:**
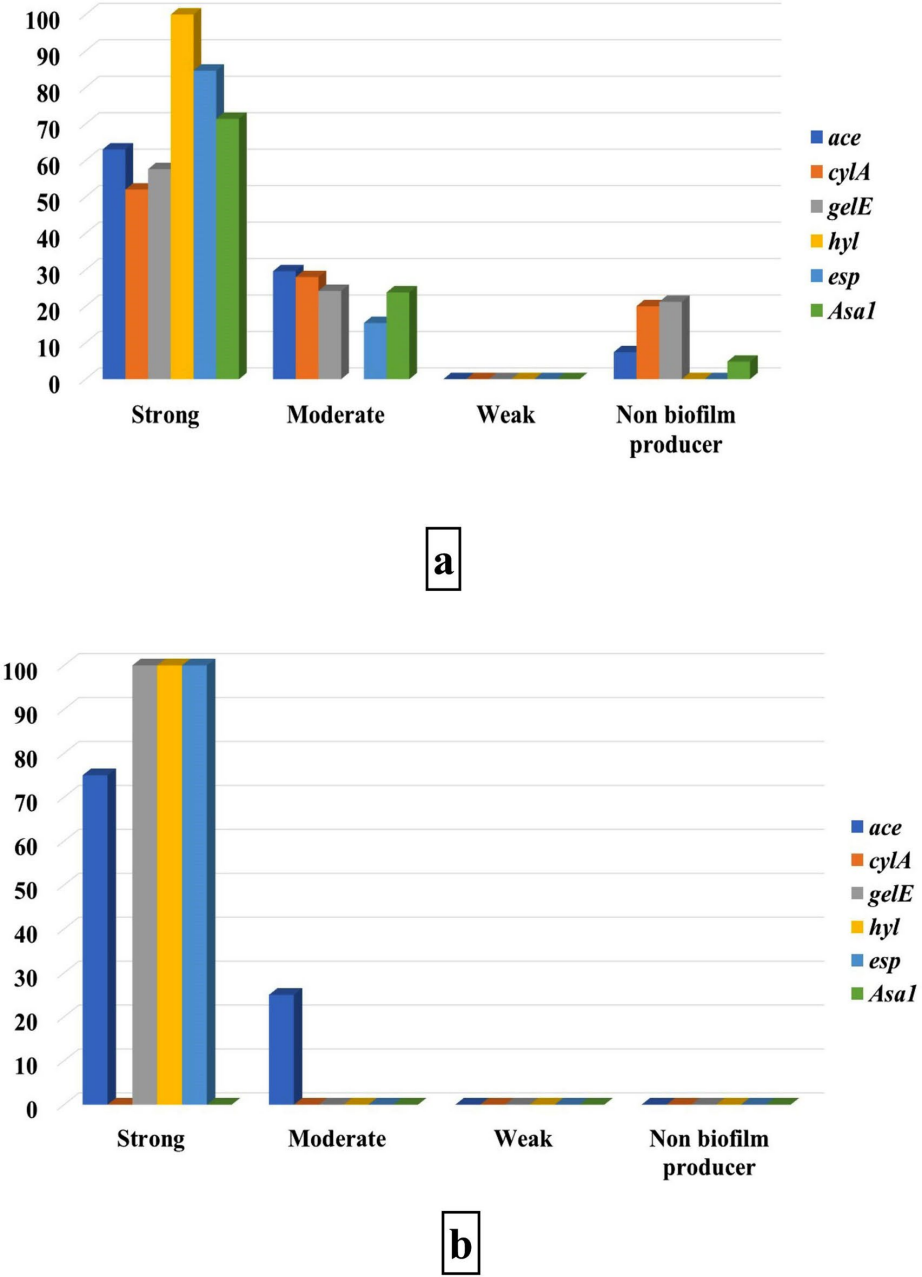
Distribution of various virulence genes among different biofilm phenotypes formed by *E. faecalis* (**a**) and *E. faecium* (**b**).

Among the 46 MDR, XDR, and PDR *E. faecalis* and *E. faecium* isolates, 56.5, 19.7, and 6.5% of the isolates, produced strong, moderate, and weak biofilm, respectively, while 17.4% were non biofilm producers (Fig. 7a). There is a strong correlation between drug resistance and biofilm production (*P* < 0.0001) and between both MDR and XDR isolates, and strong and moderate biofilm types (*P* < 0.05). Moreover, most *E. faecalis* and *E. faecium* isolates produced strong biofilms (Fig. 7b). Strongly biofilm-producing *E. faecalis* strains harbored all virulence genes with different incidences: 63, 52, 57.6, 100, 84.6, and 71.4% for *ace*, *cylA*, *gelE*, *hyl*, *esp,* and *asa1*, respectively; while these genes respectively detected in 29.6, 28, 24.2, 0, 15.4, and 23.8% of moderate biofilm-producing strains. At the same time, non-biofilm producers lack the presence of *hyl* and *esp* genes only and harbor the other genes at low frequency (Fig. 8a). Regarding strong biofilm-producing *E. facium,* the strains harbored 75% of the detected *ace* gene, while the one isolate that carried *ace*, *gelE*, *hyl,* and *esp* genes produced strong biofilm (Fig. 8b). There is a significant relation between the existence of each of *ace*, *cylA*, *gel*, *esp,* and *asa1* virulence genes and biofilm production (*P* < 0.0001), particularly *ace* and *esp genes* strongly related to strong biofilm formation by the obtained isolates.

## Discussion

The current survey evaluated the occurrence, antimicrobial resistance, and virulence factors characterization of *E. faecalis* and *E. faecium* in fish and vegetables as an unconventional source of human infection with enterococci. The findings showed that the examined fish were contaminated mainly with *E. faecalis* (12.93%, 15/116) when no *E. faecium* could be detected. The contamination rate was greater in fresh fish (19.70%) than in frozen fish (4%), which may be attributed to the adverse effect of freezing on the viability and survival of some bacteria, such as enterococci^24^. There was no significant difference in the infection rate with *E. faecalis* between the two types of fresh fish, tilapia, and catfish, which were closely related at 19.51% and 20%, respectively. Our results were consistent with various studies on the prevalence of enterococci in fish; Rahman et al.^10^ could detect *Enterococcus* species in diseased tilapia and catfish phenotypically, from which *E. faecalis* was identified by *16S rRNA* gene sequencing. Enany et al.^25^ investigated the occurrence of enterococci in tilapia fish in Ismailia, Egypt, using MALDI-TOF MS, which was 32.9%, with *E. faecalis* more prevalent than *E. faecium* using conventional PCR. Also, Nada et al.^26^ revealed that 53% of the obtained isolates from catfish harbored *EF3314*, the conserved gene of *E. faecalis*. Noroozi et al.^27^ received a higher contamination rate with *E. faecalis* in fish (30%), the highest among the examined seafood samples. A lower level of *E. faecalis* (2.7%) in farm-raised Nile tilapia was detected by El-Kader and Mousa-Balabel^28^ at Dakahlia and Kafr El-Sheikh Governorates.

Our data revealed an 11.11% (10 out of 90) contamination rate of vegetable samples with enterococci, from which *E. faecalis* was detected in 10% (9 out of 90), with the acquisition among cauliflower (16.67%). Many previous studies have agreed with us regarding the predominance of *E. faecalis* in vegetables, like Perera et al.^29^, who revealed that *E. faecalis* was most abundant in vegetables (81.6%), and Abadía-Patiño et al.^11^ who could detect *Enterococcus* species in lettuce at a percentage of 38% with the predominance of *E. faecalis*. Contrarily, a higher rate of enterococci in vegetables from markets was detected by Ben Said et al.^30^ at 84.2%, from which *E. faecium* was the most predominant detected species; they also explained that vegetables may become contaminated through soil and irrigation water, which were confirmed to be infected by enterococci in the same study.

Enterococci are an important category of bacteria that interacts complexly with humans. On the one hand, some enterococci are recognized as a natural part of human flora. However, others can cause serious illnesses in people, especially after they emerge as the third most frequent cause of nosocomial bloodstream infections^31^. In our study, we examined 120 diarrheal patients for the presence of enterococci using the culture method, which yielded 41 positive samples (34.17%) and from which, by molecular screening, a higher proportion of *E. faecalis* (23.23%; 28/120) could be identified than *E. faecium* (10.83%; 13/120). Similar results could be detected by different authors, such as Ghalavand et al.^32^ and Panpru et al.^33^. Contrarily, Özsoy, and İlki^34^ revealed a higher occurrence of *E. faecium* than *E. faecalis* in stool samples from hospitalized patients.

The present findings showed that males were more likely exposed to enterococci infection (47.83%) than females (25.68%), which agrees with Yilema et al.^35^, who reported infection rates of 6.7% and 5.7% among males and females, respectively. Incompatible findings were recorded by Abamecha et al.^36^ and Ejaz^37^, who noticed that females were more likely to be infected than males: 78.1, 74, 66.7, and 33.3%, respectively, with no statistically significant difference. This may be explained by the fact that sex differences in the occurrence of numerous infectious diseases are related to hormonal and genetic factors^38,39^. Among others, these sex differences have been described as part of the gut microbiota^40^.

In this study, the consistent predominance of enterococci infection was noticed in the young (5–17 years) and elderly (44–56 years) individuals, with percentages of 45.95 and 38.10, respectively. These findings agreed with Abamecha et al.^36^ and Yilema et al.^35^ while disagreeing with Ejaz^37^. Regarding residence, enterococci incidence was higher in urban areas (35.44) than in rural areas (31.71%). However, Yilema et al.^35^ obtained contradictory results. Variations in the environmental conditions caused by dietary changes throughout time may account for shifts in the prevalence of enterococcus communities^41^ or alterations in health status or antibiotic use^42^.

The ability of *E. faecalis* to establish itself, adhere, penetrate, evade the host's defenses, and create biofilms is essential to its pathogenesis. This important characteristic helps the bacteria survive in hostile environments^43^. This study found six virulence genes, namely *ace, cylA, gelE*, *Hyl*, *esp,* and *asa1,* in *E. faecalis* isolates obtained from food and human samples. It is clear from the obtained results that *gelE* is the dominant gene in food and human isolates, and this agrees with Todokoro et al.^44^ and Zarzecka et al.^45^. A lower incidence of the *gelE* gene in human isolates was reported by Gulhan et al.^46^ (69.2%), but when compared to the Brazilian and Bulgarian isolates, higher frequencies of the *gelE* and *cylA* genes were recorded^47,48^. The gelE gene is believed to increase enterococci's survival ability in an extraintestinal environment^49^.

There was no evidence that the *esp* gene was found among *E. faecalis* fish isolates, while one of the vegetable isolates carried the gene, with its presence in human isolates at 42.86%. Araújo et al.^50^ couldn’t detect the *esp* gene in the Brazilian research isolates; contrary, other authors recovered that gene in the Egyptian fish isolates, as did Zeid et al.^51^. On the other hand, Enany et al.^25^ found it in both fish and human isolates (72.2%). It was demonstrated that the incidence of the *esp* gene varied by country^52^. Regarding the *hyl* gene, it couldn’t be detected among vegetables and human isolates and was carried only by one fish isolate, which was consistent with the finding of research reporting that the *hyl* gene was absent in all analyzed *E. faecium* and *E. faecalis* species^53^, but contradicts what was obtained by Enany et al.^25^, who found that fish and human isolates carried that gene (19.4%). According to Sun et al.,^54^, new functions of aggregation substance )AS( encoded by *asa1* have been discovered to cause enterococcal pathogenicity. Higher occurrence rates of this gene in *E. faecalis* were reported by previous researchers^46,55,56^, unlike Kafil et al.^57^, who confirmed its absence from *E. faecalis* isolates.

Compared to food, *E. faecalis* isolated from human samples had a higher frequency of these virulence genes. This finding is consistent with the findings of^58,59^, who reported that *E. faecalis* obtained from vegetables and the environment possessed fewer determinants than clinical isolates. Therefore, the occurrence of virulence genes in *Enterococcus* species appears to be considerably different depending on origin, as Zarzecka et al.^45^ and Smoglica et al.^60^ revealed.

Moreover, the most frequent virulence gene in *E. faecium* strains isolated from humans is *ace* (30.77%), followed by *esp* (23.08%) and equal percentages of *gelE* and *hyl* (7.69). This is by the results of Bourdon et al.^61^, who described the presence of *esp* in *E. faecium* clinical isolates in France. Also, Arshadi et al.^62^ stated that *E. faecium* obtained from clinical and environmental sources harbored *esp* and *hyl* genes, referring to the role of *the esp* gene in forming a biofilm. In somewhat concordance with our results, Haghi et al.^63^ found that *ace* and *esp* were the dominant genes detected in *E. faecium* from hospitalized patients. In contrast, the investigation conducted by Aung et al.^64^ yielded *hyl* and *esp* genes as the predominant virulence factors of *E. faecium* isolates at a time when *ace* and *gelE* were absent.

The multidrug resistance of enterococci attracts attention and clarifies their preponderance in various infections. The results highlighted the major issue of antibiotic-resistant enterococci transmission through the food chain. In our study, *E. faecalis* isolates exhibited the highest resistance rates to vancomycin and tetracycline (69.23%) and a moderate rate of resistance to linezolid (40.38%). In comparison, ampicillin showed the lowest resistance (5.77%). The highest rate of tetracycline resistance was observed among diarrheal isolates (78.57%), with a moderate level of linezolid (42.86%) and no resistance to ampicillin. While *E. faecalis* isolates from vegetables showed an elevated rate of vancomycin resistance (77.78%), comparable observations by Ghalavand et al.^32^ detected tetracycline and ampicillin in stool isolates (76.2% and 0%, respectively), except for vancomycin and linezolid, which showed no resistance.

Regarding *E. faecium*, most isolates showed resistance to tetracycline (76.92%), followed by erythromycin (69.23%), with moderate resistance to vancomycin (46.15%), while the lowest resistance was for linezolid (23.08%). A Higher rate of vancomycin resistance among *E. faecium* isolates (54.2%) was observed by Özsoy and İlki^34^. According to Cai et al.^65^, *E. faecalis* and *E. faecium* exhibited superior resistance to linezolid at 58.5% and 42.3%, respectively. Khalil et al.^66^ stated tetracycline was the majority resistance among *E. faecalis* (86.36%). Still, with less resistance to vancomycin (31%), *E. faecium* isolates showed complete resistance to tetracycline and erythromycin, and both strains showed complete susceptibility to linezolid. From an epidemiological perspective, vancomycin resistance is regarded as the most serious problem related to the antibiotic resistance of enterococci^67,68^; in the U.S.A., vancomycin-resistant enterococci caused about 55,000 hospitalizations, of which more than 5,000 deaths occurred^69,70^. Also, different authors referred to the stability of vancomycin-resistant enterococci against various adverse environments, including a large temperature range (10–45 °C), a broad pH range (4.6–9.9), 40% bile salts, and 6.5% NaCl, emphasizing the greater mortality rate caused by them in cases of bloodstream infections than vancomycin-susceptible *enterococci*^71,72^. It is noteworthy that 7 out of 13 AMR patterns involving vancomycin were linezolid resistant, in addition to presenting an issue as one of the most important drugs for infections caused by multidrug-resistant enterococci, especially vancomycin-resistant Enterococci.

It is worth noting that antibiotic resistance isn’t restricted to clinical enterococci isolates, as various authors obtained it from different environmental isolates like seafood, vegetables, and other environmental samples^73–75^, which was confirmed in our study through the detection of MDR and XDR *E. faecalis* isolates derived from fish, vegetables, and humans. An important concern in hospital settings is that a patient with gastrointestinal colonization with enterococci and receiving therapeutic procedures, including antibiotic therapy, can be a source of drug-resistant enterococcal isolates, commonly resulting in an enterococcal outbreak^76^.

Numerous species of microorganisms have been documented to develop biofilms on the surface of equipment in food processing plants. Consequently, foods such as fish, meat, and vegetables can be contaminated with these microorganisms through contact with contaminated surfaces^77^. According to the obtained results, 60% of the obtained *E. faecalis* and *E. faecium* isolates were biofilm producers. Higher rates were detected by Igbinosa and Beshiru^78^ and Külahcı and Gündoğan^79^ as 67.8 and 94.2% of the obtained *Enterococcus* species, respectively, with a higher incidence for moderate biofilm producers than the strong ones.

Regarding biofilm production by *E. faecalis*, similar results were obtained by Rana et al.^80^, who detected *E. faecalis* in cultured fish samples with the ability to produce biofilm in most of them. Still, the degree of biofilm formation differed from ours in that a higher incidence of moderate biofilm producers and a lower incidence of strong biofilm producers were recorded. Additionally, 16% and 50% of *E. faecalis* were recorded as strong and intermediate biofilm producers, respectively, in a study conducted by Cui et al.^81^.

The ability of *E. faecalis* to establish itself, adhere, infiltrate, evade the host's defenses, and create biofilms is essential to its pathogenesis, which encourages the bacteria to survive in hostile environments^43^. Amongst human *E. faecalis* and *E. faecium*, 57.1 and 46.2% of fecal isolates produced biofilm, which was lower than the results recorded by Tsikrikonis et al.^49^ and Woźniak-Biel et al.^82^. In our investigation, it was found that *E. faecalis* had a higher rate of biofilm development than *E. faecium*. This agrees with Creti et al.^83^, who found that *E. faecalis* produced more biofilm than other *Enterococcus* species, regardless of their source. Also, the results reported by Necidova et al.^84^ and El-Zamkan and Mohamed^85^ showed that *E. faecalis* was more likely to develop the biofilm with its varied degree of intensity.

The present study detected a strong association between drug resistance and biofilm production. Among the different drug resistance patterns, 86.5 and 66.6% of *E. faecalis* and *E. faecium*, respectively, were able to form biofilm, which is higher than the results obtained by Gomes et al.^53^, whereas 72.4% of *E. faecalis* among antibiotic-resistant isolates were able to form biofilm. Also, in a study performed by El-Zamkan and Mohamed^85^, it was found that, compared to *E. faecium*, which contributed 20% of the moderate and strong biofilm of M.D.R. strains, *E. faecalis* isolates accounted for 44%. It is generally observed that biofilm-associated infections are a leading cause of morbidity and death^86^. According to recent research, biofilm is a hotspot for the spread of antibiotic resistance, thus developing multi-resistant strains^87^.

Findings from the current study revealed that biofilm formation depends on the presence of the *esp* and *gel*s genes. Different studies have presented conflicting results on whether the biofilm formation process depends on the existence of the *esp* gene. Some researchers have found that the *esp* gene does not play a significant role in biofilm creation^85,88^. In contrast, others have demonstrated a positive relationship between the development of biofilms and the incidence of *esp*^89,90^. Furthermore, in line with the findings of this study, Hancock and Perego^91^ found that gelatinase production is responsible for transmitting signals that trigger biofilm formation through the quorum-sensing F.S.R. system. Also, El-Zamkan and Mohamed^85^ recorded a highly significant correlation between the ability of the obtained isolates to produce biofilm and the presence of the *gelE* gene; however, Mohamed and Murray^92^ findings indicated that there is no significant relationship between the activity of gelatinase and the formation of biofilm in a considerable number of *E. faecalis* isolates. Moreover, Chajecka-Wierzchowska et al.^93^ and Igbinosa and Beshiru^78^ demonstrated that biofilm development occurred independently of *esp* and *gel* elements.

## Materials and methods

### Ethical declaration

The protocol was approved by the New Valley Research Ethics Committee (No. 02/3/11–2023/4), and informed consent was provided by all participants. The study was conducted in conformity with the ARRIVE (Animals in Research: Reporting In Vivo Experiments) criteria^94^. All methods were performed according to the relevant guidelines and regulations.

### Study design and sampling

This study was conducted in Qena Governorate, Egypt, which is approximately 576 km from Cairo (26°10′12″ N, 32°43′38″ E). The food samples included in this study were 116 fish and 90 vegetable samples, which were collected from various markets at random in Qena, Egypt, and transported in ice boxes to the laboratory immediately for bacteriological analysis.

For each fish sample, 25 g of the fish’s interior flesh and 25 g of each vegetable were blended for 2 min in 225 ml of Brain Heart Infusion broth (HiMedia, India).

Concurrently, 120 diarrheal samples from patients who approached the clinical labs in Qena suffering from diarrhea were used in the human survey. The sex, age, and residence of each participant were recorded. The samples were collected in sterile screw-capped tubes containing 5 ml broth.

### Isolation of *Enterococcus* species

Inoculated Brain Heart Infusion broth was incubated at 37 °C for 24 h, then streaked on Bile Aesculin Azide Agar plates (Oxoid, CM0888) and incubated for 24 h at 37 °C. Additionally, colonies were identified by the catalase test, Gram staining, and growth in NaCl 6.5% broth to the genus level^95^.

### D.N.A. extraction

The A.B.T. bacterial D.N.A. Mini kit (applied biotechnology, ABT001, Korean) was utilized to extract genomic DNA from enterococci cultures and was stored at − 20 °C until used.

### PCR amplification

The primers obtained from Metabion (Germany) were used in this study to identify *E. faecalis* (F: GTT TAT GCC GCA TGG CAT AAG AG; R: CCG TCA GGG GAC GTT CAG) and *E. faecium* (F: CGG TTC ATA CGGAAT GGC ACA; R: AAG TTC ACG ATA AGC CAC GG) (Additional File 2, Table S1). Primers were used in a 25-µl reaction containing 12.5 µl DreamTaq Green PCR Master Mix (2X) (Thermo Scientific, Cat No. K1081), one µl of each primer of 20 pmol concentration, 5.5 µl of grade water, and 5 µl of D.N.A. template. Agarose gel 1% was prepared in 100 ml of T.B.E. buffer. The negative control, positive control, and 20 μl of each PCR product were loaded onto the gel. The power supply ranged from 1 to 5 V/cm Gel Pilot 100 bp plus ladder (cat. no. 239045) was supplied from QIAGEN (U.S.A.). After nearly 30 min, the run was stopped, and the gel was transferred to a U.V. cabinet. Finally, the gel was photographed using a gel documentation system, and computer software was used to analyze the data.

### 16S rDNA sequencing and phylogenetic analysis

The *16S rRNA* gene PCR products for seven *E. faecalis* isolates, including tilapia fish (TF7), catfish (CF4), frozen fish (FF5 and FC6), vegetables (VS2), and diarrhea (HD1 and HS3), were purified employing the QIAquick extraction kit (Qiagen, Valencia, CA). The Bigdye Terminator V3.1 cycle sequencing kit (Perkin-Elmer) was used to sequence the resulting refined products using an Applied Biosystems 3130 Sequencer (A.B.I., U.S.A.). The sequence reaction was purified using Centrisep (spin column). The obtained sequences were uploaded to Genbank's Basic Local Alignment Search Tool (BLAST^®^). The MegAlign module of Lasergene DNAStar version 12.1 was used to construct the phylogenetic tree^96^, and phylogenetic analysis was implemented using the maximum likelihood and neighbor-joining method in MEGA 7^97^.

### Molecular detection of virulence genes

Isolates were checked for *ace, cylA, gelE*, *hyl*, *esp,* and *asa1* virulence genes (Additional File 2, Table S2). PCR amplification was performed in a 25 μL reaction mixture using 12.5 μL EmeraldAmp Max PCR Master Mix (Takara, Japan), one μL of each 20 pmol concentration primer, 4.5 μL water, and a 6 μL D.N.A. template. An Applied Biosystem 2720 thermal cycler was used for amplification, and the amplified D.N.A. was analyzed by 1.5% agarose gel electrophoresis (Applichem, Germany, GmbH).

### Phenotypic antimicrobial resistance

The antimicrobial sensitivity testing of the isolated *E. faecalis* and *E. faecium* for eight antibiotics was completed using the disc diffusion method according to the Clinical and Laboratory Standards Institute (CLSI). All *Enterococcus* isolates were tested against the major categories of antimicrobials based on their importance in human medicine. A 24-h broth cultures adjusted to 0.5 McFarland standard was inoculated to Muller Hinton agar and tested for Ampicillin (AMP, 10 μg), vancomycin (VAN, 30 μg), erythromycin (ERY, 15 μg), tetracycline (TET, 30 μg), ciprofloxacin (CIP, 5 μg), chloramphenicol (CHL, 30 μg), linezolid (LZD, 30 μg), and nitrofurantoin (NIT, 300 μg) disks. As per the guidelines provided by the Clinical Laboratory Standards Institute (CLSI)^98^, inhibitory zone diameters were calculated and classified as either sensitive or resistant.

### Biofilm formation

The biofilm production was assessed according to Hatt and Rather^99^ using microtiter plates (MTP) in triplicates. Twenty μL of each overnight grown isolate in Tryptic Soy Broth (T.S.B.) containing 1% glucose at 37 °C were transported to three wells of sterile 96-well polystyrene microtiter plates holding 180 μL of fresh T.S.B. containing 1% glucose and incubated for 24 h at 37 °C. Wells with 200 μL of uninoculated T.S.B. served as the negative control. Then, the wells were drained and washed three times with 300 μL of phosphate-buffered saline. The adhesive bacteria were then fixed with methanol for 20 min and stained with 2% crystal violet for 15 min followed by a twice thorough washing with distilled water and drying in the air. For 30 min, the dyed adherent cells were resolubilized in 150 μL of 33% acetic acid. The O.D. of each well was measured at 570 nm using a microtiter plate reader. The cut-off value (ODc) = average negative control O.D. + (3 S.D. of negative control). Accordingly, the *Enterococcus* isolates were categorized as follows: non-biofilm producers when O.D. < ODc, weak biofilm producers when ODc < O.D. < 2ODc, moderate biofilm producers when 2ODc < O.D. < 4ODc, while O.D. > 4ODc denoted strong biofilm producers as represented by Stepanovic et al.^100^.

### Statistical analysis

GraphPad Prism 8 was used for Fisher’s exact test statistical analysis of the study's data. A statistically significant result was indicated at *P* < 0.05.

## Conclusions

*E. faecalis* and *E. faecium* are adaptable zoonotic microorganisms. This research indicates that fish and vegetables are repositories for multi-resistant enterococci with virulent determinants and biofilms that may extend to humans. Regardless of the variance in the strength of biofilms among the isolates, the majority formed moderate to strong biofilms. Besides that, the isolates were also equipped with different virulence genes. From this viewpoint, consumers should be aware of the hazards accompanying consuming fresh and ready-to-eat vegetables and fish. Health and control measures must also be implemented, whether in fish farming or during irrigation of vegetables, by avoiding the use of wastewater. Also, restricted use of antibiotics in animal husbandry as well as in humans is necessary to avert a high prevalence of resistance genes.

## Supplementary Information

Supplementary Figures.

Supplementary Tables.

## Data Availability

All data analyzed during this study are available through: OR018854 https://ncbi.nlm.nih.gov/nuccore/OR018854 OR018851 https://ncbi.nlm.nih.gov/nuccore/OR018851 OR018852 https://ncbi.nlm.nih.gov/nuccore/OR018852 OR018853 https://ncbi.nlm.nih.gov/nuccore/OR018853 OR018849 https://ncbi.nlm.nih.gov/nuccore/OR018849 OR018848 https://ncbi.nlm.nih.gov/nuccore/OR018848 OR018850 https://ncbi.nlm.nih.gov/nuccore/OR018850.

## Abbreviations

*E. faecalis*: *Enterococcus faecalis*
*E. faecium*: *Enterococcus faecium*
M.D.R.: Multidrug resistance
XDR: Extensively drug-resistance
PDR: Pan drug resistance
O.D.: Optical density
