# Supplementary material for: Phenotypic and genotypic characterization of Enterococcus faecalis and Enterococcus faecium isolated from fish, vegetables, and humans
Source: Sci Rep. 2024 Sep 18;14:21741. doi: 10.1038/s41598-024-71610-0 (PMC11408632; doi:10.1038/s41598-024-71610-0)
Supplement: Supplementary file 1 — Supplementary Figures. [file 41598_2024_71610_MOESM1_ESM.docx]

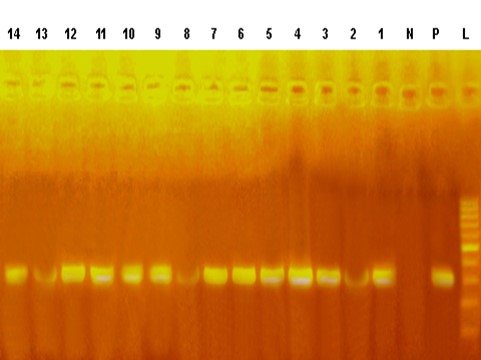


**Figure S1:** Agarose gel electrophoresis of *E. faecalis 16S rRNA* gene in isolates from different examined samples showing bands at 310 bp. Lane L: DNA ladder (100 bp), Lane P: positive control, Lane N: negative control, Lanes 1, 2, 3, 4, 5, 6, 7, 8, 9, 10, 11, 12, 13, 14: positive *E. faecalis 16S rRNA* gene.


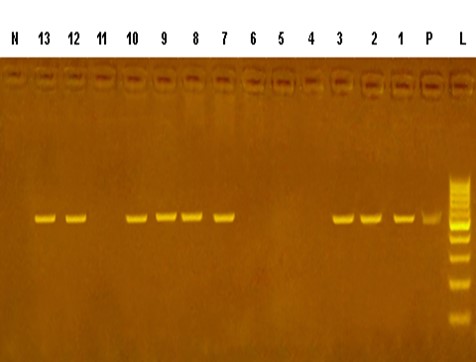


**Figure S2:** Agarose gel electrophoresis of *E. faecium atpA* gene in isolates from different examined samples showing bands at 556 bp. Lane L: DNA ladder (100 bp), Lane P: positive control, Lanes 1, 2, 3, 7, 8, 9, 10, 12, 13: positive *E. faecium atpA* gene, and Lanes 4, 5, 6, 11: negative for the gene. Lane N: negative control.


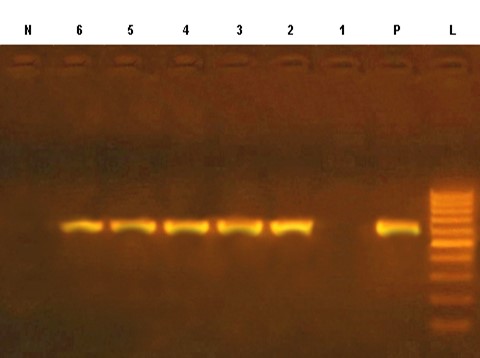


**Figure S3:** Agarose gel electrophoresis of *Ace* gene in the obtained *E. faecalis* and *E. faecium* isolates showing bands at 616 bp. Lane L: DNA ladder (100 bp), Lane P: positive control, Lane 1: negative for *ace* gene, Lanes 2, 3, 4, 5, 6: positive for the gene, and Lane N: negative control.


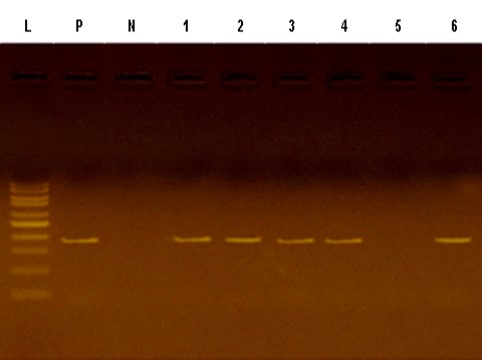


**Figure S4:** Agarose gel electrophoresis of *Asa1* gene in the obtained *E. faecalis* and *E. faecium* isolates showing bands at 375 bp. Lane L: DNA ladder (100 bp), Lane P: positive control, Lane N: negative control, Lanes 1, 2, 3, 4, 6: positive for *Asa1* gene, and Lane 5: negative for the gene.


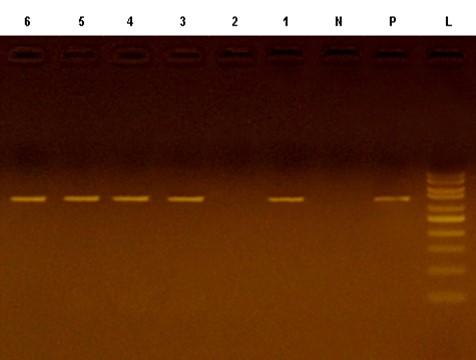


**Figure S5:** Agarose gel electrophoresis of of *cylA* gene in the obtained *E. faecalis* and *E. faecium* isolates showing bands at 688 bp. Lane L: DNA ladder (100 bp), Lane P: positive control, Lane N: negative control, Lanes 1, 3, 4, 5, 6: positive for *cylA* gene, Lane 2: negative for *cylA* gene.


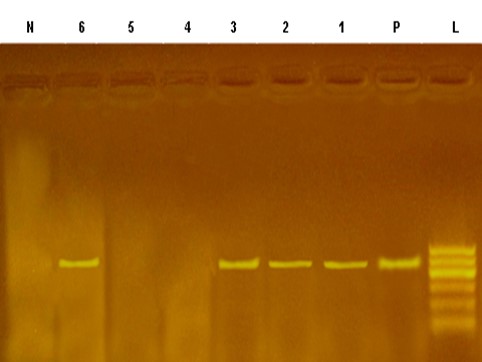


**Figure S6:** Agarose gel electrophoresis of *Esp* gene in the obtained *E. faecalis* and *E. faecium* isolates showing bands at 510 bp. Lane L: DNA ladder (100 bp), Lane P: positive control, Lanes 1, 2, 3, 6: positive for *Esp* gene, Lanes 4, 5: negative for the gene, Lane N: negative control.


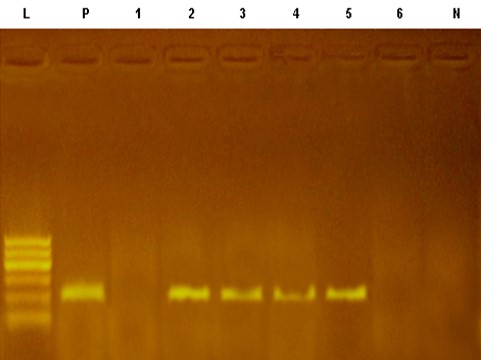


**Figure S7:** Agarose gel electrophoresis of *gelE* gene in the obtained *E. faecalis* and *E. faecium* isolates showing bands at 213 bp. Lane L: DNA ladder (100 bp), Lane P: positive control, Lanes 1, 6: negative for *gelE* gene, Lanes 2, 3, 4, 5: positive for the gene. Lane N: negative control.


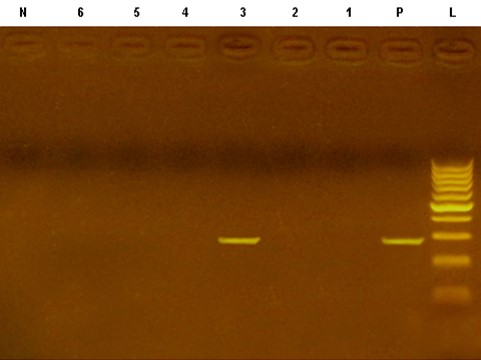


**Figure S8:** Agarose gel electrophoresis of *Hyl* gene in the obtained *E. faecalis* and *E. faecium* isolates showing bands at 276 bp. Lane L: DNA ladder (100 bp), Lane P: positive control, Lanes 1, 2, 4, 5, 6: negative for *Hyl* gene, Lane 3: positive for the gene. Lane N: negative control.
