# Supplementary material for: Phenotypic and genotypic characterization of Enterococcus faecalis and Enterococcus faecium isolated from fish, vegetables, and humans
Source: Sci Rep. 2024 Sep 18;14:21741. doi: 10.1038/s41598-024-71610-0 (PMC11408632; doi:10.1038/s41598-024-71610-0)
Supplement: Supplementary file 2 — Supplementary Tables. [file 41598_2024_71610_MOESM2_ESM.docx]

**Table S1. Primers sequences of E. faecalis and E. faecium genes, amplicon sizes and cycling conditions.**

| **Target gene** | **Primers sequences** | **Amplified segment (bp)** | **Primary**  **denaturation** | **Amplification (35 cycles)** | | | **Final extension** | **Reference** |
| --- | --- | --- | --- | --- | --- | --- | --- | --- |
|  |  |  |  | **Secondary denaturation** | **Annealing** | **Extension** |  |  |
| ***E. faecalis 16S rRNA*** | GTT TAT GCC GCA TGG CAT AAG AG | 310 | 94˚C  5 min | 94˚C  30 sec | 50˚C  30 sec. | 72˚C  30 sec | 72˚C  7 min. | [1] |
|  | CCG TCA GGG GAC GTT CAG |  |  |  |  |  |  |  |
| ***E. faecium atpA*** | CGG TTC ATA CGGAAT GGC ACA | 556 | 94˚C  5 min | 94˚C  30 sec | 50˚C  40 sec | 72˚C  45 sec | 72˚C  10 min. | [2] |
|  | AAG TTC ACG ATA AGC CAC GG |  |  |  |  |  |  |  |

1. Zoletti GO, Siqueira JF, Santos KRN. Identification of Enterococcus faecalis in Root-filled Teeth With or Without Periradicular Lesions by Culturedependent and—Independent Approaches. JOE 2006; 32: 8
2. Homan WL, Tribe D, Poznanski S, Li M, Hogg G, Spalburg E, van Embden JDA, Willems RJL. Multilocus Sequence Typing Scheme for *Enterococcus faecium.* JOURNAL OF CLINICAL MICROBIOLOGY 2002; 40(6):,1963–1971.

**Table S2. Primers sequences of virulence genes, amplicon sizes and cycling conditions.**

| **Target gene** | **Primers sequences** | **Amplified segment (bp)** | **Primary**  **denaturation** | **Amplification (35 cycles)** | | | **Final extension** | **Reference** |
| --- | --- | --- | --- | --- | --- | --- | --- | --- |
|  |  |  |  | **Secondary denaturation** | **Annealing** | **Extension** |  |  |
| ***Ace*** | GGAATGACCGAGAACGATGGC | 616 | 94˚C  5 min. | 94˚C  30 sec. | 58˚C  40 sec. | 72˚C  45 sec. | 72˚C  10 min. | [3] |
|  | GCTTGATGTTGGCCTGCTTCCG |  |  |  |  |  |  |  |
| ***cylA*** | ACTCGGGGATTGATAGGC | 688 | 94˚C  5 min. | 94˚C  30 sec. | 50˚C  40 sec. | 72˚C  45 sec. | 72˚C  10 min. | [4] |
|  | GCTGCTAAAGCTGCGCTT |  |  |  |  |  |  |  |
| ***gelE*** | TATGACAATGCTTTTTGGGAT | 213 | 94˚C  5 min. | 94˚C  30 sec. | 50˚C  30 sec. | 72˚C  30 sec. | 72˚C  7 min. |  |
|  | AGATGCACCCGAAATAATATA |  |  |  |  |  |  |  |
| ***Hyl*** | ACAGAAGAGCTGCAGGAAATG | 276 | 94˚C  5 min. | 94˚C  30 sec. | 55˚C  30 sec. | 72˚C  30 sec. | 72˚C  7 min. |  |
|  | GACTGACGTCCAAGTTTCCAA |  |  |  |  |  |  |  |
| ***Esp*** | AGATTTCATCTTTGATTCTTGG | 510 | 94˚C  5 min. | 94˚C  30 sec. | 50˚C  40 sec. | 72˚C  45 sec. | 72˚C  10 min. |  |
|  | AATTGATTCTTTAGCATCTGG |  |  |  |  |  |  |  |
| ***Asa1*** | GCACGCTATTACGAACTATGA | 375 | 94˚C  5 min. | 94˚C  30 sec. | 50˚C  40 sec. | 72˚C  45 sec. | 72˚C  10 min. |  |
|  | TAAGAAAGAACATCACCACGA |  |  |  |  |  |  |  |

1. Creti R, Imperi M, Bertuccini L, Fabretti F, Orefici G, Di Rosa R, et al. Survey for virulence determinants among *Enterococcus faecalis* isolated from different sources. J Med Microbiol. 2004;53: 13–20. doi:10.1099/jmm.0.05353-0
2. Vankerckhoven V, Van Autgaerden T, Vael C, Lammens C, Chapelle S, Rossi R, et al. Development of a multiplex PCR for the detection of *asa1*, *gelE*, *cylA*, *esp*, and *hyl* genes in *Enterococci* and survey for virulence determinants among European hospital isolates of *Enterococcus* *faecium*. J Clin Microbiol. 2004;42: 4473–4479. doi:10.1128/JCM.42.10.4473-4479.2004
